# Rectal Endometriosis-Associated Adenocarcinoma: A Rare Entity Demanding Clinical Vigilance

**DOI:** 10.7759/cureus.78661

**Published:** 2025-02-07

**Authors:** Xiao-Hai Huang, Yu-Chieh Weng, Yang-Bor Lu

**Affiliations:** 1 Department of Digestive Disease, Xiamen Chang Gung Hospital, Hua Qiao University, Fujian, CHN

**Keywords:** colonoscope, colorectal cancer, endometrioid adenocarcinoma, endometriosis-associated, s: endometriosis

## Abstract

Endometriosis may become malignant, and its diagnosis is challenging, especially in rare extra-ovarian sites like the rectum. Here, we describe the case of a 42-year-old woman who presented with intermittent left lower abdominal discomfort for six months. A colonoscopy revealed a 1.5 × 2 cm rectal lesion of high-grade intraepithelial neoplasia based on endoscopic biopsy. During surgical exploration, the mass was noted to be adherent to the posterior uterine wall, prompting partial rectal resection, total hysterectomy, and bilateral salpingectomy. Histopathological evaluation revealed a poorly differentiated adenocarcinoma infiltrating the rectum and posterior uterine myometrium, with benign endometriotic foci contiguous to the malignant component. Immunohistochemical staining supported a diagnosis of endometriosis-associated adenocarcinoma involving the rectum.

Given its rarity and clinical mimicry of primary colorectal cancer, rectal endometriosis-associated adenocarcinoma presents a significant diagnostic challenge. This case underscores the importance of comprehensive surgical evaluation and detailed histopathological and immunohistochemical analyses to distinguish such tumors from primary colorectal malignancies and to guide optimal therapeutic strategies.

## Introduction

Endometriosis is a common gynecological proliferative disease that affects 5-15% of reproductive-age women worldwide [1] and has the potential for malignant transformation with locally aggressive behavior [2]. It is characterized by endometrial-like tissue located outside the uterine cavity, most frequently in the ovaries. However, extra-ovarian, such as in the fallopian tubes, uterosacral ligaments, and even beyond the pelvis (including the rectum), are rare but can still occur [3]. Although endometriosis is not presumed to be a clinical premalignant condition, it possesses the capacity for malignant change [4]. Indeed, the incidence of malignant transformation in endometriosis ranges from 0.7 to 1.0%, and about only one-quarter of these malignancies arise from extra-ovarian implants [5]. It can develop into various histological subtypes, including endometrioid adenocarcinoma (the most common subtype) or clear cell carcinoma, whereas endometrial stromal sarcomas, adenosarcomas, and carcinosarcomas are very rare [6]. Because endometriosis-associated adenocarcinoma in the rectum is uncommonly observed, particularly when typical endometrial carcinoma symptoms, such as irregular vaginal bleeding and pelvic pain, are absent, it makes a definite diagnosis challenge. In this report, we describe a patient without any endometriosis-related clinical manifestations who presented with a rectal lesion (8 cm from the anal verge), which was initially diagnosed as high-grade intraepithelial neoplasia, without a medical history of endometriosis, thereby underscoring the need for heightened clinical vigilance for this uncommon entity.

## Case presentation

**Figure 1:**
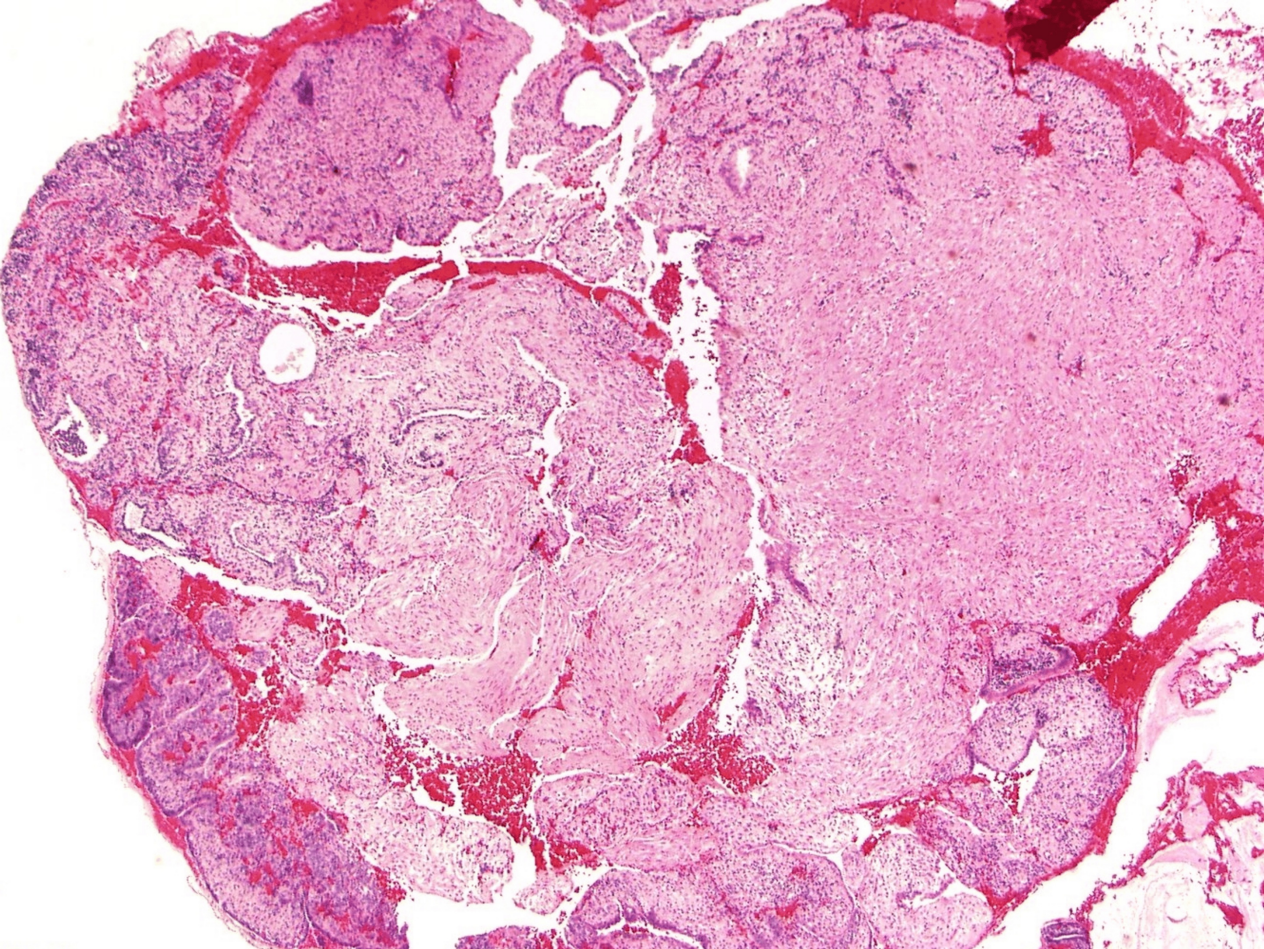
Histology of uterine polyp. Polypoid fragments of endometrial tissue lined by epithelium (H&E, 40×). H&E, hematoxylin and eosin stain

A 42-year-old reproductive female presented with a chief complaint of intermittent left lower abdominal discomfort for six months. She denied bowel habit alternations, hematochezia, or irregular vaginal bleeding. Her past medical history was notable for uterine polyp (Figure 1), for which she underwent polypectomy the previous year. On physical examination, her abdomen was soft and non-tender, and rectal examination revealed no visible bleeding nor palpable mass. Laboratory parameters were within normal ranges, indicating a leukocyte count of 6.14 × 10^9^/L (normal range, 3.5-9.5 × 10^9^/L) and hemoglobin of 135 g/L (normal range, 115-150 g/L). Serum carcinoembryonic antigen (CEA) was 0.62 ng/mL (normal, <5 ng/mL), cancer antigen 19-9 (CA 19-9) was 9.06 U/mL (normal, 0-28), and CA 125 was 27.2 U/mL (normal, <35 U/mL).

**Figure 2:**
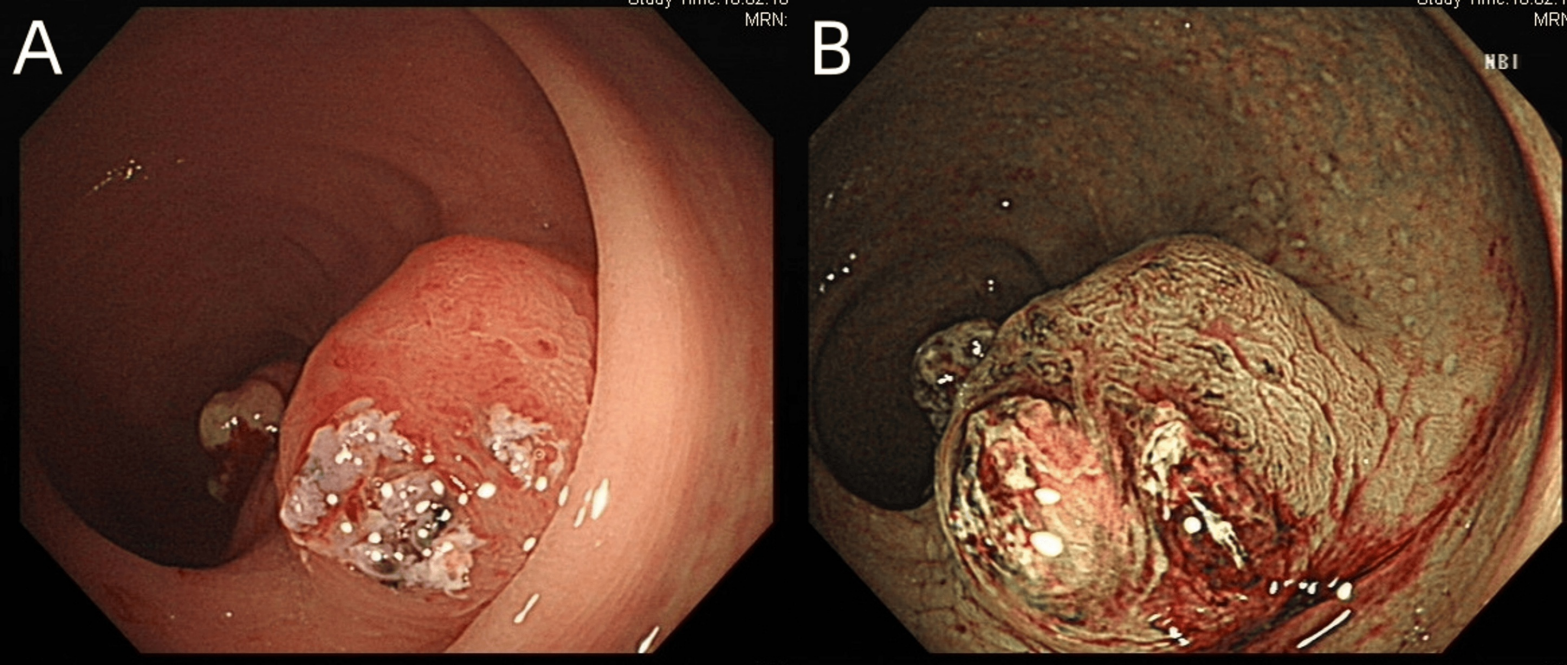
Colonoscope finding. A 1.5 × 2 cm lesion that appeared centrally ulcerated, fungating, and fleshy. (A) White light endoscopy. (B) Narrow band imaging.

**Figure 3:**
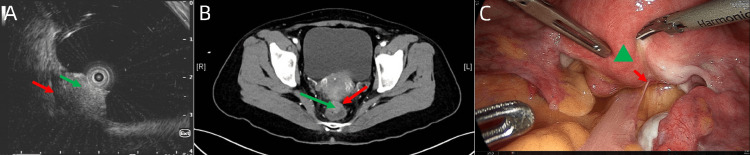
Patient’s images (endoscopic ultrasound). (A) Endoscopic ultrasound demonstrated a tumor (green arrow) invading the muscular layer of the rectum (red arrow). (B) An abdominal CT demonstrated an exophytic nodular 1.7 × 2.1 cm mass narrowing the colonic lumen (green arrow) with an intact colonic serosa (red arrow). (C) Intraoperative photo shows the posterior uterine wall (green triangle) was invaded by the tumor (red arrow).

Colonoscopy identified a 1.5 × 2 cm lesion located in the rectum 8 cm from the anal verge, which appeared centrally ulcerated, fungating, and fleshy (Figure 2). Endoscopic ultrasound (EUS) demonstrated a 2 cm mass with poorly defined borders, suggesting invasion into the muscular layer (Figure 3A). An abdominal computed tomography (CT) demonstrated an exophytic nodular 1.7 × 2.1 cm mass narrowing the colonic lumen, with an intact colonic serosa and no apparent adenopathy (Figure 3B), supporting a presumptive diagnosis of colorectal cancer. Endoscopic biopsy of the lesion revealed high-grade intraepithelial neoplasia, highly suggestive of adenocarcinoma. Based on these findings, the patient was provisionally diagnosed with primary colorectal adenocarcinoma and underwent a low anterior resection (Dixon operation) to preserve the anal sphincter. Intraoperatively, the tumor was found to adhere to the uterine posterior wall (Figure 3C). Following multidisciplinary consultation with a gynecologist and a thorough examination of the uterus and bilateral adnexa, the surgical plan was extended to include partial rectal resection, total hysterectomy, and bilateral salpingectomy.

**Figure 4:**
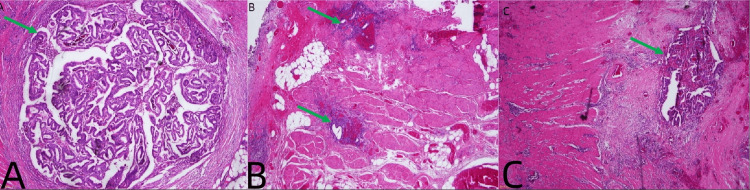
Pathological features. (A) Histological section showing endometrioid adenocarcinoma with irregular glandular structures infiltrating the intestinal wall (green arrow, H&E, 40×). (B) Benign endometriosis in the rectal muscular layer (green arrow, H&E, 100×). (C) Atypical hyperplasia between the benign and malignant endometrial tissues could be observed (green arrow, H&E, 100×). H&E, hematoxylin and eosin stain

**Figure 5:**
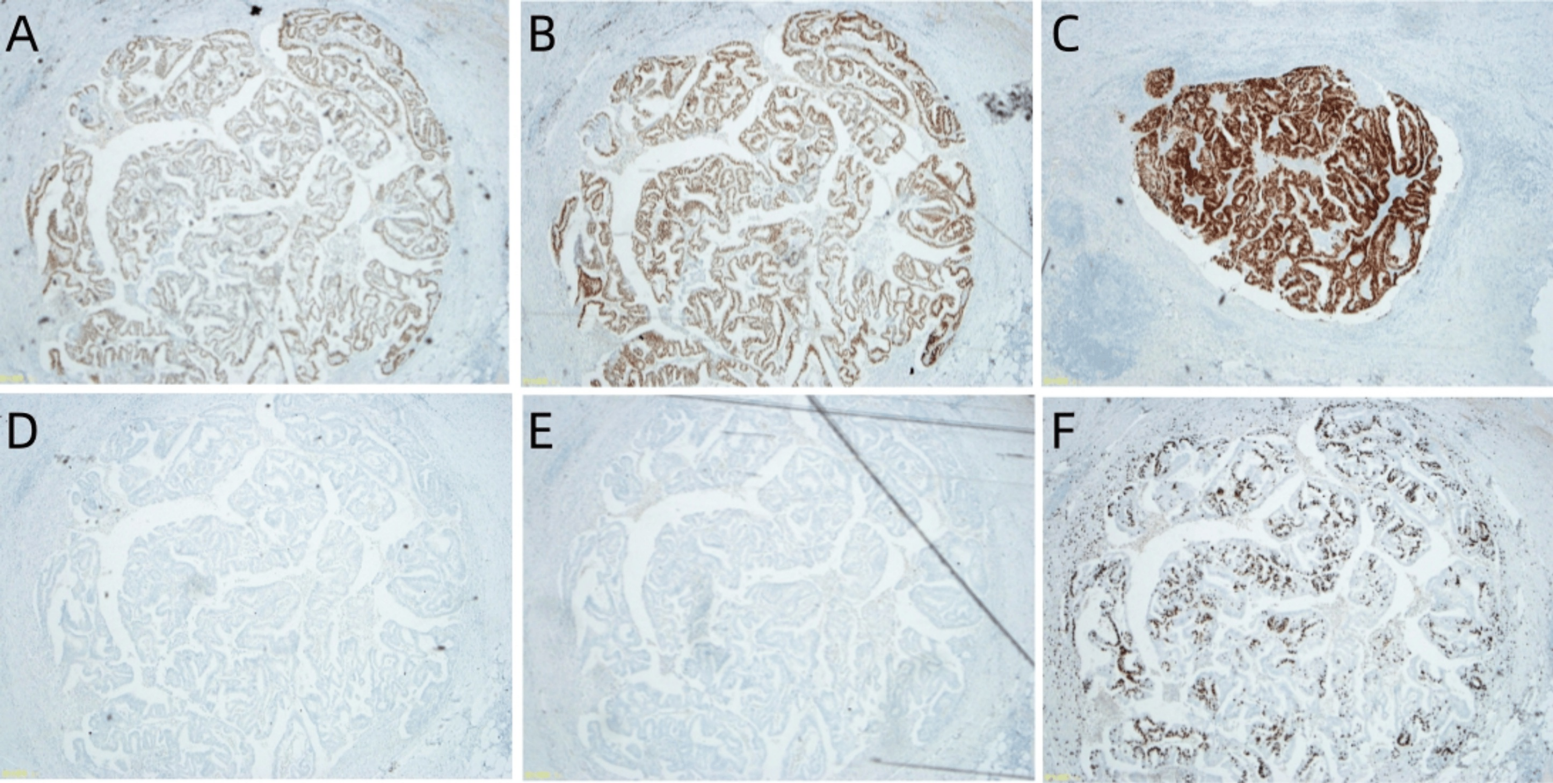
Immunohistochemical stains. The tumor cells were positive for (A) estrogen receptor (ER, 40×); (B) progesterone receptor (PR, 40×); (C) paired-box gene 8 (PAX8, 40×). While the tumor cells were negative for (D) caudal type homeobox 2 (CDX2, 40×); (E) Special AT-rich sequence-binding protein 2 (SATB2, 40×); and (F) 70% for Ki-67 index (40×).

A gross examination revealed a 3.0 × 2.0 × 0.7 cm mass involving the rectum and infiltrating into the posterior uterine myometrium. No additional primary tumors were identified in the endometrium, cervix, or bilateral fallopian tubes. Histologically, the neoplasm displayed poor glandular differentiation consistent with adenocarcinoma (Figure 4A), and none of the 32 resected lymph nodes were involved. Microscopically, benign endometriosis foci were noted in the muscular layer of the rectum (Figure 4B), contiguous with an endometroid adenocarcinoma infiltrating all layers of the rectum and the posterior uterine myometrium (Figure 4C). Immunohistochemistry staining (IHCs) revealed that the tumor was positive for estrogen receptor (ER; Figure 5A), progesterone receptor (PR, Figure 5B), and paired-box gene 8 (PAX 8) (Figure 5C). At the same time, caudal type homeobox 2 (CDX2; Figure 5D) and special AT-rich sequence-binding protein 2 (SATB2; Figure 5E) were absent. The Ki-67 index was 60% (Figure 5F). 

These IHC findings confirmed a diagnosis of endometriosis-associated adenocarcinoma of the rectum, an unexpected occurrence. The patient recovered uneventfully and was subsequently started on adjuvant chemotherapy with paclitaxel (Taxol) and carboplatin. 

## Discussion

This case with ENZIAN(s) [7] P0; O0/0; T0+/0+; A2; B0/0; C2; FX/ stage 1 of revised American Society for Reproductive Medicine (rASRM) with score 4 [8], that fulfilled the criteria for the diagnosing malignant transformation of rectal endometriosis [9] including, 1) coexistence of both malignant and benign endometrial tissues within the tumor; 2) histological features consistent with endometrial origin; 3) absence of other primary tumor sites; and 4) demonstration of a histological transition from benign to malignant endometriosis. Despite meeting these criteria, it remains challenging to differentiate an endometriosis-associated adenocarcinoma involving the intestinal wall from a primary colorectal adenocarcinoma. Such tumors can invade the bowel wall from the peritoneal cavity between the uterus and rectum, progressing through the serosa, subserosa, muscularis propria, and, ultimately, the mucosa [10]. In another way, they may arise from the ectopic endometrial tissue within the intestinal wall and extend outward, affecting adjuvant layers, including the mucosa. In either scenario, the lesion may mimic the intraepithelial neoplasia of primary colorectal carcinoma when evaluated by endoscopic biopsy [11], such as our presented case. Thus, growth patterns alone cannot conclusively determine whether a tumor originates from the bowel itself or presents a malignant transformation of ectopic endometrial tissue. Morphological assessment can be misleading, highlighting the need for supplementary diagnostic modalities such as IHCs to accurately establish the tumor’s origin. Moreover, no other primary tumors were found in the endometrium of the hysterectomy and ovarian specimen.

In addition, a previous report has demonstrated that high microsatellite instability (MSI-high) and mutations in genes, such as KRAS, PIK3CA, and PTEN, are associated with rectal endometriosis-associated adenocarcinoma [12]. These molecular features are not commonly seen in typical colorectal adenocarcinomas. Thus, while we did not directly assess these gene tests, their reported association supports the hypothesis that the rectal tumor in our case could have arisen from endometriosis.

Rectal endometriosis-associated carcinoma is exceedingly rare, and its etiology remains uncertain. Sporadic case series have been reported [12], the majority of which involved patients with a history of pelvic surgery (e.g., hysterectomy for endometriosis [13] or myomectomy [14]) performed years prior to cancer development. In our patient, a previous uterine polypectomy may have facilitated the dissemination of endometrial tissue and increased the risk of malignant transformation.

While both rectal endometriosis-associated endometrioid adenocarcinoma and primary rectal adenocarcinoma may share overlapping treatment modalities, distinguishing between them is crucial. Unlike rectal adenocarcinoma, which has well-established guidelines [15], there is currently no consensus on the standard therapeutic approach for rectal endometriosis-associated malignancies. However, primary cytoreductive surgery should be performed whenever possible to achieve complete resection of all detectable lesions. Although most reported patients have been treated with a combination of surgery and individualized chemotherapy, the exact benefit of chemotherapy remains uncertain.

## Conclusions

Rectal endometriosis-associated carcinoma is rare yet a distinct clinical entity. Careful distinction from colorectal carcinoma is essential to guide optimal therapeutic approaches. Clinicians should remain vigilant, especially in patients with a history of pelvic surgery, given the potential for endometriotic lesions to undergo malignant transformation.
